# Acupuncture for patients with myasthenia gravis: A systematic review protocol

**DOI:** 10.1097/MD.0000000000017563

**Published:** 2019-10-18

**Authors:** Shuai Shi, Xinyu Ji, Yanping Wang, Bin Liu, Huamin Zhang, Cheng Lu

**Affiliations:** a Guang’anmen Hospital; b Institute of Basic Research in Clinical Medicine; c Institute of Information on Traditional Chinese Medicine, China Academy of Chinese Medical Sciences, Beijing, China.

**Keywords:** acupuncture, myasthenia gravis, protocol, randomized controlled trials, systematic review

## Abstract

**Background:**

The objective of this systematic review protocol is to provide the methods for evaluating the effectiveness and safety of acupuncture on the treatment of myasthenia gravis (MG).

**Methods and analysis:**

We will search randomized controlled trials (RCTs) on this subject in 8 electronic databases and they are Cochrane Central Register of Controlled Trials (CENTRAL), MEDLINE, Embase, PubMed, Chinese Biomedical Literature Database (CBM), China National Knowledge Infrastructure (CNKI), the Wan-Fang Database, and Chinese Scientific Journal Database (VIP database). Other relevant literatures will be manually searched as a complement. Only RCTs related to acupuncture for MG will be included, without Language restrictions and limitation of publication types. The risk of bias and trial quality will be assessed by the Cochrane collaboration tool. The study inclusion, data extraction and quality assessment will be conducted independently by 2 reviewers. All data from the studies included will be analyzed by RevMan V.5.3 statistical software.

**Results:**

This study will provide a high-quality synthesis of RCTs on the efficacy and safety of acupuncture as an adjuvant therapy in the treatment of MG.

**Conclusion:**

This systemic review will provide high quality evidence to evaluate acupuncture as adjuvant therapy in patients with MG.

**Prospero registration number:**

PROSPERO CRD42019133577.

## 1 Introduction

Myasthenia gravis (MG) is an autoimmune antibody-mediated disorder characterized by fluctuating fatigability and weakness affecting ocular, bulbar, and limb skeletal muscle groups.^[1]^ The total MG incidence and prevalence is 5.3 per million person-years and 77.7 cases per million of the population, respectively.^[2]^ And mortality is up to approximately 40%.^[3]^ At present, the incidence and prevalence of MG are increasing, particularly in older individuals.^[2,4]^ According to a large clinical study of unselected patients with MG in China, the most striking result is the high proportion of childhood cases mostly with purely ocular MG, which is different from clinical expression of caucasian with MG.^[5]^ Ocular weakness, the most common initial presentation of MG, occur in approximately 85% of patients.^[6]^ According to a large number of MG epidemiological studies, the prevalence of fatigue was 70%.^[7]^ Various clinical treatments for MG exist, including thymectomy, symptomatic, and immunosuppressive (IS) treatments, and immunomodulating therapies such as intravenous immunoglobulin (IVIg) and plasma exchange (PLEX).^[8,9]^

However, there is no internationally accepted standard of care, and no one treatment best for all patients because of heterogeneous of MG.^[9,10]^ Standards and possibilities for the diagnosis and treatment of myasthenia gravis show great variation within and between countries.^[11]^ In particular, orthodox therapy for effective symptom control often requires prolonged and even life-long IS treatment with high-dose steroids and add-on other IS agents.^[12–14]^ Furthermore, despite there have been significant advances in the treatment of MG, an estimated 10% to 20% of patients with MG do not achieve an adequate response, are intolerant to conventional treatment.^[15]^ In addition, the adverse effects associated with these treatments are significant, such as diarrhea, nausea, vomiting, salivating, muscle twitching, and the treatments suffer from short effectiveness, difficult dosage control, strong dependence, and high cost.^[16]^ Implementing best-practice standards universally represents a major challenge.^[11]^

Complementary and alternative medicine (CAM) is increasingly used to treat MG, owing to its long-term efficacy and few side effects. Acupuncture is one of the most frequently used forms of complementary medicine.^[17]^ Nowadays, an increasing number of patients with MG seek help from complementary and alternative medicine. Acupuncture, an important part of traditional Chinese medicine (TCM), has been used to treat MG diseases for a long time and obtained experimental evidence.^[18]^ According to our pre-search, many clinical trials, which were conducted to investigate the efficacy of acupuncture for patients with MG, indicated that acupuncture could relieve the patients’ symptoms. However, there has been no systematic evaluation of the safety and efficacy of acupuncture in the treatment of myasthenia gravis. Thus, we conducted a systematic review of acupuncture for MG focused on the clinical evidence according to the high-quality randomized-controlled clinical trials (RCTs).

## 2 Objectives

This systematic review aims to analyze various RCTs, further summarize and critically evaluate the evidence for the effectiveness and safety of acupuncture treatment of MG.

## 3 Methods

This protocol of this systematic review has been registered on PROSPERO, the registration number is CRD42019133577. The protocol will be strictly developed under the guidelines of Preferred Reporting Items for Systematic Reviews and Meta-Analyses protocols (PRISMA-P).^[19]^

### 3.1 Eligible criteria for study selection

#### 3.1.1 Types of studies

We will only include RCTs of acupuncture treating MG without publication or language restriction. And quasirandomized RCTs will be ruled out, such as case report, and the study without sufficient information about the randomized method or process.

#### 3.1.2 Types of participants

We will include patients with any sex, age, nationality, and education background, who are diagnosed with myasthenia gravis. Myasthenia gravis diagnoses are on the grounds of the internationally recognized criteria, such as the diagnosis criteria settled by the Myasthenia Gravis Foundation of America (MGFA),^[20]^ China guidelines for the diagnosis and treatment of myasthenia gravis.^[21]^ The other diagnostic criteria with comparable definitions were also used.

#### 3.1.3 Types of interventions

##### 3.1.3.1 Experimental interventions

Clinical trials with all types of acupuncture intervention including regular acupuncture, electroacupuncture, fire needling, scalp needling, auricular acupuncture, catgut embedding acupuncture, and intradermal needling will be included. While we will exclude other forms of stimulation including laser acupuncture, cupping, moxibustion, bleeding therapy, acupotomy, pharmaco acupuncture, or point injection.

##### 3.1.3.2 Control interventions

Eligible control group will include the studies that are interfered with no treatment, sham acupuncture, placebo or Western drug. Additionally, studies compare acupuncture plus the concomitant of other treatment with that treatment will also meet the control group including criteria.

#### 3.1.4 Types of outcome measures

##### 3.1.4.1 Primary outcomes

We will consider the following outcomes measuring the extent of MG and muscle weakness:

(1) Quantitative Myasthenia Gravis (QMG) scores.^[22]^
(2) MG clinical absolute and relative scores or other validated scales.^[23]^ The MG clinical absolute and relative scores are a 60-point scale evaluate ptosis, eyelid fatigue, eye movement in the horizontal direction, right and left arm held outstretched at 90°, flexion of the knee and hip at 90°, facial muscles, chewing and swallowing, and respiratory muscle function.
(3) Clinical absolute score before and after treatment.
(4) Clinical relative score and effective rate: The clinical relative score = (absolute score before treatment – absolute score after treatment)/absolute score before treatment 100%.

##### 3.1.4.2 Secondary outcomes

The secondary outcomes included:

(1) Relapse rate after follow-up.
(2) Adverse events.
(3) Acetylcholine receptor antibody (AchRAb): The concentration of AchRAb in Serum.
(4) Quality of life. Such as MG activities of daily living (ADL) profile (MG-ADL)-a simple 8-question survey of MG symptoms.^[24]^

### 3.2 Search strategy

Two reviewers independently searched the following databases: PubMed, EMBASE, Cochrane Central Register of Controlled Trials (CENTRAL), Cochrane Library, Chinese Biomedical Literature Database, China National Knowledge Infrastructure (CNKI), Chinese Scientific Journal Database (VIP) Journals Database, Wan fang data Information Site without language restriction. The search time was from inception to September 30th of 2019. The reference lists of retrieved trials and previous systematic reviews will be searched for citation of potentially eligible trials. We will contact the author of articles if there were any question about trials.

In addition, we will search the WHO International Clinical Trials Registry Platform (http://www.who.int/trialsearch) the US National Institutes of Health Ongoing Trials Register (http://www.clinical.trials.gov) and meta Register of Controlled Trials (http://www.controlledtrials.com) for any unpublished or relevant ongoing trials. The following search terms will be used: myasthenia gravis, acupuncture, manual acupuncture, electroacupuncture, fire needling, auricular acupuncture, ear acupuncture, dermal needle, abdominal acupuncture, pyonex and plum blossom needle, controlled clinical trial, randomized, placebo, randomly. The search strategy for PubMed is shown in Table 1, which will be modified if necessary. The reference lists of the relevant articles will also be checked.

**Table 1:**
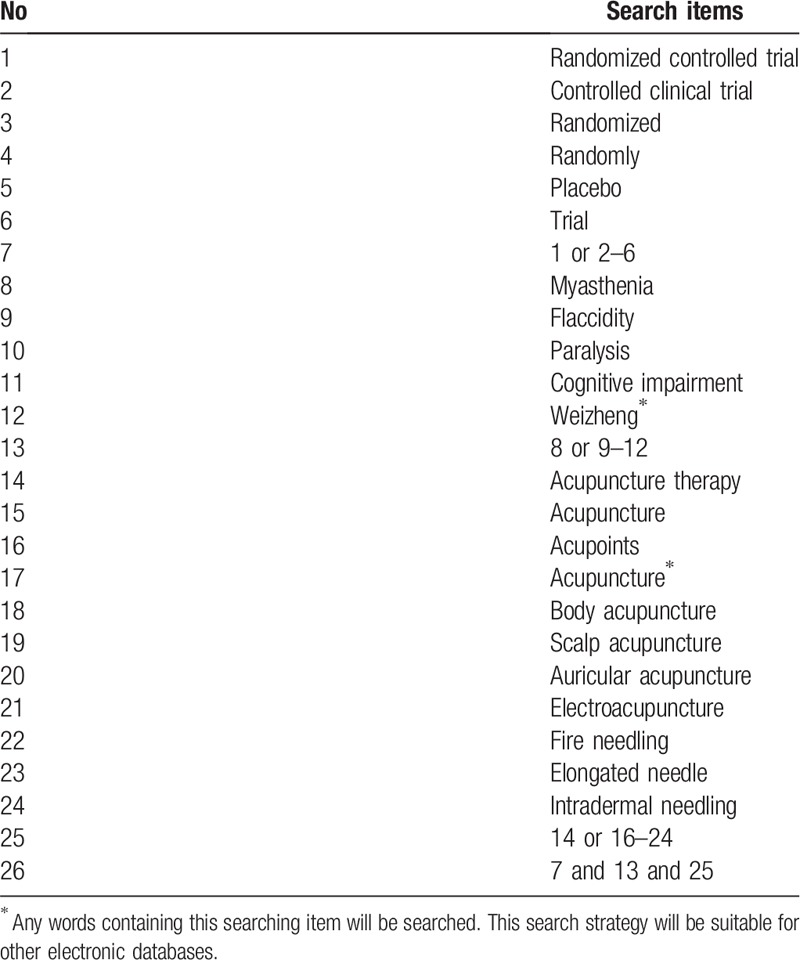
Search strategy used in PubMed database.

#### 3.2.1 Searching other resources

Additionally, the following Chinese medical journals will also be searched as complement: Chinese Acupuncture and Moxibustion (1985–2019), Acupuncture Research (1985–2019), China Journal of Traditional Chinese Medicine and pharmacy (1985–2019). Meanwhile, relevant conference papers will be manually retrieved. At the same time, we will also search the WHO International Clinical Trials Registry Platform (ICTRP) to contain new trails pertaining to the theme.

### 3.3 Data collection and analysis

#### 3.3.1 Study selection

**Figure 1:**
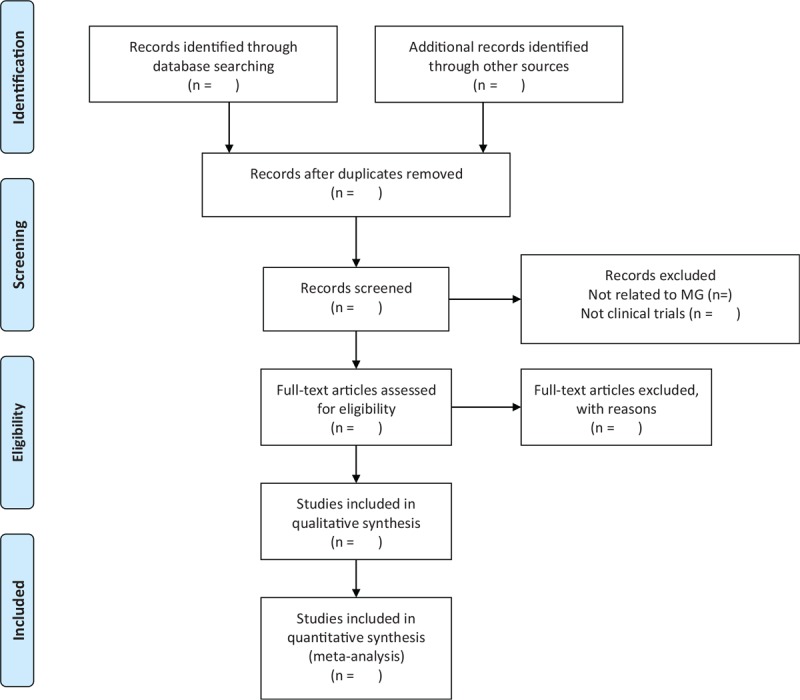
PRISMA flow diagram of study process. PRISMA = preferred reporting items for systematic reviews and meta-analyses.

After electronic searches in the databases, 2 reviewers (SS and JXY) will screen the titles and abstracts respectively to exclude: the duplicates; the studies in which the participants in the experimental group did not receive acupuncture treatment as the primary intervention; the studies that were not RCTs with parallel design; and the studies in which the participants did not meet the criteria of MG. Then our reviewers will screen the full text articles that cannot be obviously screened by titles and abstracts only. And reviewers will analysis consideration to identify the included studies and assess the study eligibility. Reasons for the exclusion of studies will be recorded. In addition, all reviewers will have a group discussion on the consistency of all the studies included, exclude and eliminate those are not up to the theme topic till final team consensus arrived. The selection process is fully elucidated in the following PRISMA flow diagram (Fig. 1).

#### 3.3.2 Data extraction and management

An electronic form will be established via Excel to extract substantial contents, and then filled by 2 reviewers independently. Information was extracted from the qualified articles by using a standardized data extraction form as follows: general information: first author, the year of publication, and country; participants: sample size, sex, age, and disease duration; methodological characteristics: study design, MG severity, and diagnostic criteria; details of intervention: type of intervention, duration of treatment, and follow-up time; and outcome measures. Disagreements will be solved by group discussion or consult seniors. However, if we fail to reach the consensus, the authors of trials will be contacted for further details and verification.

#### 3.3.3 Assessment of bias risk and quality of included studies

Two independent authors assess the risk of bias of each included article according to the Cochrane Handbook for Systematic Reviews of Interventions. The methodological quality will be evaluated from the following domains: generation of random sequence, allocation concealment, blinding of participants and personnel, blinding of outcome assessments, incomplete outcome data, selective reporting, and other bias. Description of each aspect of risk of bias in each pooled study will be conducted in order to provide the rationale for the risk judgment. The risks will be classified into 3 levels: low, high, or unclear with a graphical presentation.

#### 3.3.4 Measurements of treatment effect

Statistical analysis will be performed by Cochrane Collaboration Review Manager Software (RevMan 5.3). Standard chi-squared test and *I*^2^ statistic will be used to examine the heterogeneity between trial and control results. A fixed effects model (*I*^2^ < 50%) or a random effects model (*I*^2^ > 50%) will be used depending on the value of *I*^2^. The value of *P* < .05 was considered statistically significant. Dichotomous outcomes were calculated by the risk ratio (RR) with 95% confidence interval (CI), whereas continuous outcomes will be summarized using standardized mean difference (SMD) with 95% CI. Publication bias will be checked graphically by using the funnel plot, and approximately symmetric shows no existence of publication bias.

#### 3.3.5 Managing missing data

If any data information is not sufficient in included trials, we will try to contact the first or corresponding author by email or phone, requesting for adequate information and details of the studies included to retrieve missing or insufficient trial data. However, if the author is not available or sufficient information cannot be obtained, we will have a group discussion and analysis based on the current information. Meanwhile, the potential impact of missing data will be taken into account and relative discussion will be presented in the result section.

#### 3.3.6 Assessment of heterogeneity

Based on the guidelines in Cochrane Handbook for systematic review, we will perform a standardized chi-squared test (*α* = 0.1) and *I*^2^ value, respectively, to assess the heterogeneity. If *I*^2^ ≤ 50%, the studies included will indicate no presence of meaningful heterogeneity, and then a fixed-effect model will be employed to evaluate the effect sizes. While, if *I*^2^ > 50%, indicating the heterogeneity among studies are statistical significant, and a random-effect model will be adopted.

#### 3.3.7 Assessment of reporting biases

We will use funnel plots to detect reporting biases and small-study effects. When the number of trials included in the meta-analysis is >10, a visual asymmetry on the funnel plot will be developed to evaluate the existing bias of included studies.

#### 3.3.8 Data synthesis

The synthesis will be carried out using RevMan V.5.3 provided by Cochrane Collaboration. In accordance to Cochrane guideline, if *I*^2^ < 50%, a fix-effect model will be employed to evaluate mean difference and relative risk. Otherwise, the source of heterogeneity will be analyzed using a random-effect model to exclude obvious clinical heterogeneity. If evident heterogeneity is found between studies, we will conduct a subgroup analysis to explore the possible reasons attributing to this statistical heterogeneity, and present a reasonable explanation.

#### 3.3.9 Subgroup analysis

To detect the heterogeneity between groups under the condition that the existing studies are sufficient, we will conduct a subgroup analysis to explore the feasibility of the review conclusions. The subgroup analysis will be carried out to interpret the robustness of studies according to following aspects: sex and age of patients, different forms of acupuncture intervention (needles, frequency, tensity, points, duration, and treatment session).

#### 3.3.10 Sensitivity analysis

After removing the low-quality studies, we will perform a sensitivity analysis to evaluate the robustness of the results according to following aspects: sample size, missing data, and methodologically quality. The sensitivity analysis is conducted to exclude the inappropriate trials without random generation.

#### 3.3.11 Grading the quality of evidence

The Grading of Recommendations Assessment, Development and Evaluation (GRADE) guidelines will be employed to evaluate the quality of evidence for primary outcomes. The strength of evidence will be graded it into very low, low, moderate, or high level.

#### 3.3.12 Dissemination and ethics

Formal ethical approval is not required in this protocol. We will collect and analyze data based on published studies, and since there is no patients involved in this study, individual privacy will not be under concerns. The results of this review will be disseminated to peer-reviewed journals or submit to related conferences.

## 4 Discussion

MG brings a significant adverse impact on patients’ daily activities, including quality of life, general and psychological health. Pharmacological methods have been associated with a slight increase in incidence of adverse effects and surgery is not appropriate for each patient. Acupuncture therapy, a suggested intervention in China that may have value to treat MG, has been shown to improve some symptoms of myasthenia gravis, such as eye weakness and fatigue. But no high-quality synthesis of the evidence exists. To our knowledge, there is no related systematic review published in English. Therefore, a high quality systematic review is needed. And we show the process of performing this study in Fig. 1, which will be divided into 4 parts, including identification, selection, data extraction and management, and data analysis. We hope that this systematic review will provide the current clinical evidence on the effectiveness and safety of acupuncture treatment for MG, and analyze which type of myasthenia gravis suitable for acupuncture treatment. Those can provide more useful information to the doctor in clinical practice and better choice for patients.

This study may have limitations that might limit its ability to generate conclusions based on high confidence. Specifically, there may be significant heterogeneity in the forms of acupuncture therapies used and the qualities of methodology. There will also most likely be differences in outcomes measured and tools used.

## Author contributions

LC, ZHM, WYP conceived this study and developed the first frame of this manuscript. JXY, SS, drafted the manuscript. LB, JXY performed data collection; SS revised the manuscript. All authors contributed to the editing of the final manuscript and approved the final version.
